# Ferroptosis, mitochondrial stress and redox imbalance: a pharmacological-toxicological biochemistry framework for precision drug therapeutics

**DOI:** 10.3389/fphar.2026.1890015

**Published:** 2026-10-22

**Authors:** Okechukwu Paul-Chima Ugwu, Chinyere Nneoma Ugwu, Godson Emeka Anyanwu, Mariam Basajja, Maryann Chiamaka Ebuoh

**Affiliations:** 1 Department of Research, Publication and Extension, Kampala International University, Kampala, Uganda; 2 Department of Anatomy, Faculty of Biomedical Sciences, Kampala International University, Kampala, Uganda; 3 Department of Human Anatomy, State University of Medical and Applied Sciences, Igbo Eno, Enugu, Nigeria

**Keywords:** drug toxicity, ferroptosis, lipid peroxidation, mitochondrial electron transport chain, pharmacological biochemistry, precision therapeutics, redox imbalance

## Abstract

Ferroptosis, mitochondrial stress and redox imbalance are increasingly recognised as interconnected determinants of therapeutic efficacy, drug resistance and organ toxicity. Ferroptosis is an iron-dependent form of regulated cell death driven by lipid peroxidation and impaired antioxidant defences, with relevance to cancer, cardiovascular disease, inflammatory disorders, ischaemia-reperfusion injury and drug-induced tissue damage. This Perspective reframes the ferroptosis-mitochondrial stress-redox axis as a unified pharmacological-toxicological platform rather than an isolated therapeutic target, resolving its mechanisms to individual electron transport chain complexes, membrane phospholipid species and downstream immunological consequences. Beyond the conventional GPX4-system Xc^−^NRF2 framework, we incorporate dihydroorotate dehydrogenase (DHODH), GCH1-BH4 signalling, NCOA4-mediated ferritinophagy and p53-dependent regulation of ferroptosis sensitivity. We further define how complex I- and III-derived superoxide, compartmentalised inner-membrane lipid pools and ACSL4/LPCAT3-directed phospholipid remodelling shape organ-specific vulnerability. Because ferroptotic cells release damage-associated molecular patterns whose effects depend on cell death kinetics, immunomodulation is considered a distinct therapeutic opportunity and safety consideration. To consolidate these interacting mechanisms into a single, comprehensible reference, we present an integrated molecular pathway diagram that maps oxidative-stress chemistry, compartment-resolved mitochondrial involvement, lipid-substrate specificity, cell-death execution and kinetics-dependent immunological crosstalk onto their corresponding pharmacological entry points. We propose a tiered biomarker framework, a p53-and biomarker-guided patient stratification strategy, a chemically stratified therapeutic map distinguishing repurposed drugs from experimental compounds, and a conceptual pharmacokinetic-pharmacodynamic framework linking therapeutic and toxic exposure windows. Future research should prioritise biomarker validation, complex-specific mitochondrial functional assays, lipidomic profiling, organ-specific toxicology, tumour-selective delivery systems and pharmacokinetic-pharmacodynamic integration to advance safer, more precise ferroptosis-based therapeutics.

## Introduction

Modern drug therapeutics increasingly depends on a careful distinction between biochemical vulnerability and toxicological liability. Ferroptosis illustrates this tension particularly well. In one setting, it can be exploited to kill therapy-resistant diseased cells; in another, it may contribute to avoidable injury in the liver, kidney, heart or brain. Dixon et al. (2012) first described ferroptosis as an iron-dependent, non-apoptotic form of regulated cell death. Since then, the field has moved rapidly, with ferroptosis now implicated in cancer, cardiovascular disease, neurodegeneration, inflammatory pathology and toxicological biochemistry (Chen et al., 2024; Dixon et al., 2012; Zhou et al., 2024).

The execution of ferroptosis depends on iron availability, lipid peroxidation, glutathione depletion, glutathione peroxidase 4 (GPX4) dysfunction, system Xc–inhibition, ferroptosis suppressor protein 1 (FSP1), coenzyme Q10 metabolism, NRF2-mediated antioxidant defence and mitochondrial redox state (Bersuker et al., 2019; Dai et al., 2024; Doll et al., 2019; Yan et al., 2023; Zhang et al., 2024). More recent work has broadened this picture beyond the canonical glutathione–GPX4 system to include dihydroorotate dehydrogenase (DHODH) at the mitochondrial inner membrane, the GTP cyclohydrolase 1 (GCH1)-tetrahydrobiopterin (BH4) antioxidant axis, NCOA4-mediated ferritinophagy as a source of labile iron, and p53 as a context-dependent modulator of ferroptosis sensitivity (Kang et al., 2024; Mao et al., 2021; Soula et al., 2020). These pathways do not operate in isolation. In practice, they form an integrated pharmacological and toxicological network that links efficacy, resistance and safety.

This is why ferroptosis is best understood not simply as a cell-death mechanism, but as a translational framework for precision therapeutics. The specific contribution of this Perspective is therefore sevenfold. First, whereas most existing reviews catalogue ferroptosis mechanisms or focus on a single therapeutic area (most often oncology), we treat induction and inhibition as two directions of one continuum and make the bidirectional efficacy–toxicity trade-off the organising principle of the analysis. Second, we integrate emerging regulators (DHODH, GCH1–BH4, NCOA4-ferritinophagy and p53) that are frequently omitted from translationally focused discussions, and resolve their mechanistic contribution to the level of individual electron transport chain complexes and compartmentalised lipid pools rather than treating mitochondrial stress as a single generic variable. Third, we specify how lipid-peroxidation substrate chemistry, principally the arachidonoyl- and adrenoyl-phosphatidylethanolamine species generated through ACSL4 and LPCAT3, dictates tissue-specific membrane vulnerability, rather than treating lipid peroxidation as a uniform event. Fourth, we treat the immunomodulatory consequence of ferroptotic cell death within the tumour microenvironment as a distinct translational axis, because the same damage-associated molecular patterns that can promote antitumour immunity in early ferroptosis can instead recruit immunosuppressive myeloid populations once cell death has progressed further. Fifth, we convert this mechanistic synthesis into actionable translational tools, namely, a chemically and pharmacokinetically stratified agent map (Table 1), a tiered candidate biomarker panel with explicit provisional validation benchmarks (Table 2), a p53-and biomarker-guided stratification framework (Table 3), and a single, comprehensive molecular pathway diagram (Figure 1) that consolidates the oxidative-stress chemistry, compartment-resolved mitochondrial biology, cell-death execution and immunological crosstalk described throughout the text, so that the cross-talk between these nodes can be read as one integrated schematic rather than reconstructed piecemeal from separate sections. Sixth, we provide a conceptual pharmacokinetic-pharmacodynamic model for reconciling overlapping therapeutic and toxic exposure windows, and we explicitly separate acute, reversible ferroptotic injury from chronic, blood-brain-barrier-constrained neurodegenerative disease, because these contexts impose materially different dosing, scheduling and delivery requirements. In doing so we aim to move ferroptosis from an attractive mechanism towards a structured, biomarker-guided and safety-aware drug-development strategy.

**TABLE 1:** Chemically and pharmacokinetically stratified pharmacological-toxicological biochemistry framework for ferroptosis-based drug therapeutics, with representative agents and disease-specific applications.

Target/process	Representative agent(s)	Chemical/PK class	Therapeutic opportunity and disease example	Toxicological concern	Translational priority
GPX4 inhibition	RSL3, ML162, altretamine	Tool compounds (RSL3, ML162): poor aqueous solubility, limited systemic PK; altretamine: approved oral alkylating agent, distinct mechanism	Induces ferroptosis in apoptosis-resistant tumours (e.g., therapy-resistant melanoma, clear-cell renal carcinoma)	Hepatic, renal, cardiac and neuronal lipid-peroxidation injury; early ferroptosis may be immunogenic (DAMP release), but late-stage debris can recruit immunosuppressive myeloid cells	Tissue-selective delivery matched to solubility class; GPX4 activity and DAMP/immune monitoring
System Xc– blockade	Erastin, IKE, sulfasalazine	Erastin/IKE: lipophilic tool compounds, poor PK; sulfasalazine: approved oral prodrug with established human PK	Depletes cystine/glutathione in redox-dependent tumours (e.g., diffuse large B-cell lymphoma, pancreatic cancer)	Oxidative injury in normal tissues with low antioxidant reserve; DAMP release with context-dependent immunogenic or tolerogenic effect	Baseline glutathione and redox-capacity assessment; PK-matched formulation
FSP1-CoQ10 pathway	iFSP1, viFSP1	Early-stage experimental tool compounds; PK not yet characterised *in vivo*	Overcomes GPX4-independent resistance (e.g., GPX4-inhibitor-tolerant lung adenocarcinoma)	Loss of lipid radical-trapping protection in normal cells	Combination safety studies and lipidomic monitoring of PE-AA/PE-AdA species
DHODH inhibition	Brequinar; teriflunomide (repurposing)	Brequinar: IV experimental agent with historical human PK data; teriflunomide/leflunomide: approved oral agents with well-characterised PK	Sensitises low-GPX4 tumours via loss of mitochondrial lipid-peroxide defence	Mitochondrial toxicity in heart, liver and kidney via loss of the sole defence against complex I/III-derived superoxide in the inner membrane; immunosuppression	Complex-resolved mitochondrial respiration assays; cardiac/hepatic monitoring
GCH1-BH4 axis	Experimental GCH1 inhibitors	Early-stage experimental tool compounds; no established human PK	Targets BH4-dependent resistance not covered by GPX4/FSP1	Disrupts nitric oxide and neurotransmitter cofactor supply	Confirm on-pathway killing; neurovascular safety profiling
NCOA4/ferritinophagy	Iron-modulating agents	Heterogeneous class spanning approved iron chelators and experimental autophagy modulators	Raises labile iron to enhance ferroptosis in iron-avid tumours	Iron-mediated injury; off-target ferritin depletion	Labile-iron-pool phenotyping; chelation-rescue studies
NRF2 signalling	Bardoxolone (activation); ML385 (inhibition)	Bardoxolone: clinical-stage oral agent with human PK data; ML385: preclinical tool compound	Protects normal tissue (activation) or resensitises tumours (inhibition)	Activation may promote cancer survival and drug resistance	Context-specific activation or inhibition
Iron chelation (protective)	Deferoxamine, deferiprone	Approved parenteral (deferoxamine) and oral (deferiprone) chelators with established human PK	Limits ferroptosis in DILI, AKI and IR injury; well matched to short, peri-procedural acute dosing	Over-chelation and systemic iron deficiency with prolonged use	Iron-status phenotyping and dose individualisation; restrict duration to the acute injury window
Radical-trapping antioxidants	Ferrostatin-1, liproxstatin-1	Lipophilic tool compounds with poor aqueous solubility and limited systemic PK; not blood-brain-barrier optimised	Protects against ferroptotic tissue injury (e.g., neurodegeneration, IR injury)	May reduce anticancer ferroptosis efficacy; peripheral chemistries are poorly suited to chronic CNS dosing	Disease-specific use; separate acute peripheral and chronic CNS delivery chemistries
Biomarker-guided dosing	Companion-diagnostic panel (Table 2)	Not applicable (diagnostic, not therapeutic, class)	Aligns therapy with ferroptosis sensitivity and redox phenotype	Poorly validated clinical thresholds	Standardised panels, provisional benchmarks (Table 2) and prospective validation

AKI, acute kidney injury; BH4 = tetrahydrobiopterin; CNS, central nervous system; CoQ10 = coenzyme Q10; DAMP, damage-associated molecular pattern; DHODH, dihydroorotate dehydrogenase; DILI, drug-induced liver injury; FSP1 = ferroptosis suppressor protein 1; GCH1 = GTP, cyclohydrolase 1; GPX4 = glutathione peroxidase 4; IKE, imidazole ketone erastin; IR, ischaemia-reperfusion; NCOA4 = nuclear receptor coactivator 4; NRF2 = nuclear factor erythroid 2-related factor 2; PE-AA/PE-AdA, arachidonoyl-/adrenoyl-phosphatidylethanolamine; PK, pharmacokinetic. Agents in parentheses are experimental or repurposing candidates rather than approved ferroptosis-directed drugs. The chemical/PK, class column is intended to make explicit that agents grouped under a shared mechanistic target may nonetheless require different formulation and delivery strategies.

**TABLE 2:** Proposed tiered ferroptosis biomarker panel, intended use, sample type, validation status and provisional benchmarks.

Tier	Biomarker(s)	Intended use/interpretation	Sample type	Validation status	Proposed provisional benchmark (hypothesis-generating)
Predictive (sensitivity)	ACSL4, GPX4, SLC7A11, FSP1, DHODH	High ACSL4 with low GPX4/SLC7A11 predicts a ferroptosis-sensitive tumour; FSP1/DHODH flag bypass resistance	Tumour tissue (IHC, RNA)	Preclinical to early translational; ACSL4 most advanced; thresholds not standardised	Provisional working ratio: ACSL4-high defined as upper tertile of IHC H-score with concurrent GPX4/SLC7A11 in the lower tertile of the same cohort, to be recalibrated per tumour type during prospective validation
Pharmacodynamic (engagement)	Oxidised PE-AA/PE-AdA, MDA, 4-HNE, lipid ROS, GSH/GSSG	Confirms on-target lipid peroxidation and antioxidant depletion after dosing, with oxidised PE-AA/PE-AdA as the ferroptosis-specific tier	Tumour biopsy, plasma	Oxidised PE-AA/PE-AdA assay-validated in research; MDA/4-HNE established but non-specific; not yet qualified as clinical PD endpoints	Provisional target: post-dose rise in oxidised PE-AA/PE-AdA of at least two-fold over paired pre-dose baseline, alongside a GSH/GSSG ratio fall of at least fifty per cent, as a working confirmation of on-target engagement pending prospective calibration
Sensitivity cofactors	Labile iron pool, NCOA4, NRF2 activation, BH4 level	Refines the sensitivity/resistance estimate and dosing	Tissue, blood	Exploratory; methods heterogeneous	No provisional numerical benchmark proposed; recommended for qualitative stratification only until assay harmonisation is achieved
Safety (organ injury)	Circulating MDA/4-HNE, ALT/AST, creatinine/cystatin C, troponin, NGAL	Early detection of hepatic, renal or cardiac ferroptotic injury	Serum/plasma	Organ-injury markers clinically established; ferroptosis specificity still being defined	Provisional alert threshold: any organ-injury marker rising above the standard clinical reference range together with a concurrent rise in circulating MDA/4-HNE, used as a composite trigger for mitochondrial functional work-up rather than either marker alone
Functional (reserve)	Mitochondrial respiration (complex-resolved), membrane potential, ATP capacity	Estimates normal-tissue vulnerability before and during therapy	Tissue, PBMCs	Research-grade; standardisation and prospective validation required	Provisional flag: complex I- or complex III-specific respiratory reserve below eighty per cent of the matched pre-treatment baseline, prompting dose review before further mitochondrially targeted dosing
Immunological (kinetics-dependent)	Circulating HMGB1, calreticulin exposure, tumour-infiltrating CD8^+^ T cells and myeloid populations	Distinguishes early, immunogenic ferroptosis from late, tolerogenic ferroptosis	Serum/plasma, tumour tissue	Exploratory; largely preclinical	No provisional numerical benchmark proposed; recommended as an exploratory co-marker pending assay standardisation

4-HNE, 4-hydroxynonenal; ALT/AST, alanine/aspartate aminotransferase; BH4 = tetrahydrobiopterin; GSH/GSSG, reduced/oxidised glutathione; HMGB1 = high-mobility group box one protein; IHC, immunohistochemistry; MDA, malondialdehyde; NGAL, neutrophil gelatinase-associated lipocalin; PBMC, peripheral blood mononuclear cell; PD, pharmacodynamic; PE-AA/PE-AdA, arachidonoyl-/adrenoyl-phosphatidylethanolamine; ROS, reactive oxygen species. No marker is yet a qualified, regulator-accepted companion diagnostic for ferroptosis. The provisional benchmarks are illustrative, hypothesis-generating starting points proposed to focus prospective validation studies; they are not derived from a completed prospective trial and must not be used to direct current clinical practice.

**TABLE 3:** Proposed p53-and biomarker-guided decision framework for ferroptosis-directed therapy selection.

Scenario	p53 status	Predictive biomarker profile (Table 2, tier 1)	Recommended action	Rationale
Favourable induction candidate	Wild-type, retained SLC7A11-repressive capacity	High ACSL4; low GPX4/SLC7A11; adequate normal-tissue reserve	GPX4 or system Xc– inducer monotherapy may be trialled, with organ-reserve monitoring per Table 2	p53-mediated SLC7A11 repression is expected to add to pharmacological system Xc– blockade, and a favourable predictive profile suggests the tumour is intrinsically sensitised (Liu and Gu, 2022; Mao et al., 2021)
p21-adapted resistance	Wild-type or mutant with evidence of p21-dependent metabolic adaptation	Intermediate or low ACSL4; preserved GPX4/SLC7A11; elevated FSP1 or DHODH	Consider combination targeting a second node (e.g., sub-toxic DHODH inhibition with GPX4 inhibition) rather than monotherapy	p21-dependent adaptation is expected to blunt single-node induction; combining nodes addresses redundant defence systems (Liu and Gu, 2022; Bersuker et al., 2019; Doll et al., 2019)
p53-null or loss-of-function	Loss of function, no SLC7A11-repressive capacity	Variable ACSL4; frequently preserved GPX4/SLC7A11	Prioritise agents acting independently of the p53-SLC7A11 axis (e.g., DHODH or FSP1-directed strategies) over system Xc– blockade	Without p53-mediated SLC7A11 repression, system Xc– blockade alone is less likely to be sufficient (Liu and Gu, 2022)
Ambiguous or intermediate profile	Status uncertain or discordant across assays	Discordant or borderline tier 1 markers	Defer induction therapy; obtain further tissue profiling (Table 2, tiers 1-3) before selecting an agent	Acting on a discordant predictive profile risks either under-treating a resistant tumour or exposing normal tissue to unnecessary toxicity

This framework is a provisional, hypothesis-generating stratification rule intended to make the context-dependence of p53 operational for trial design; it requires prospective validation before informing individual clinical decisions.

**FIGURE 1:**
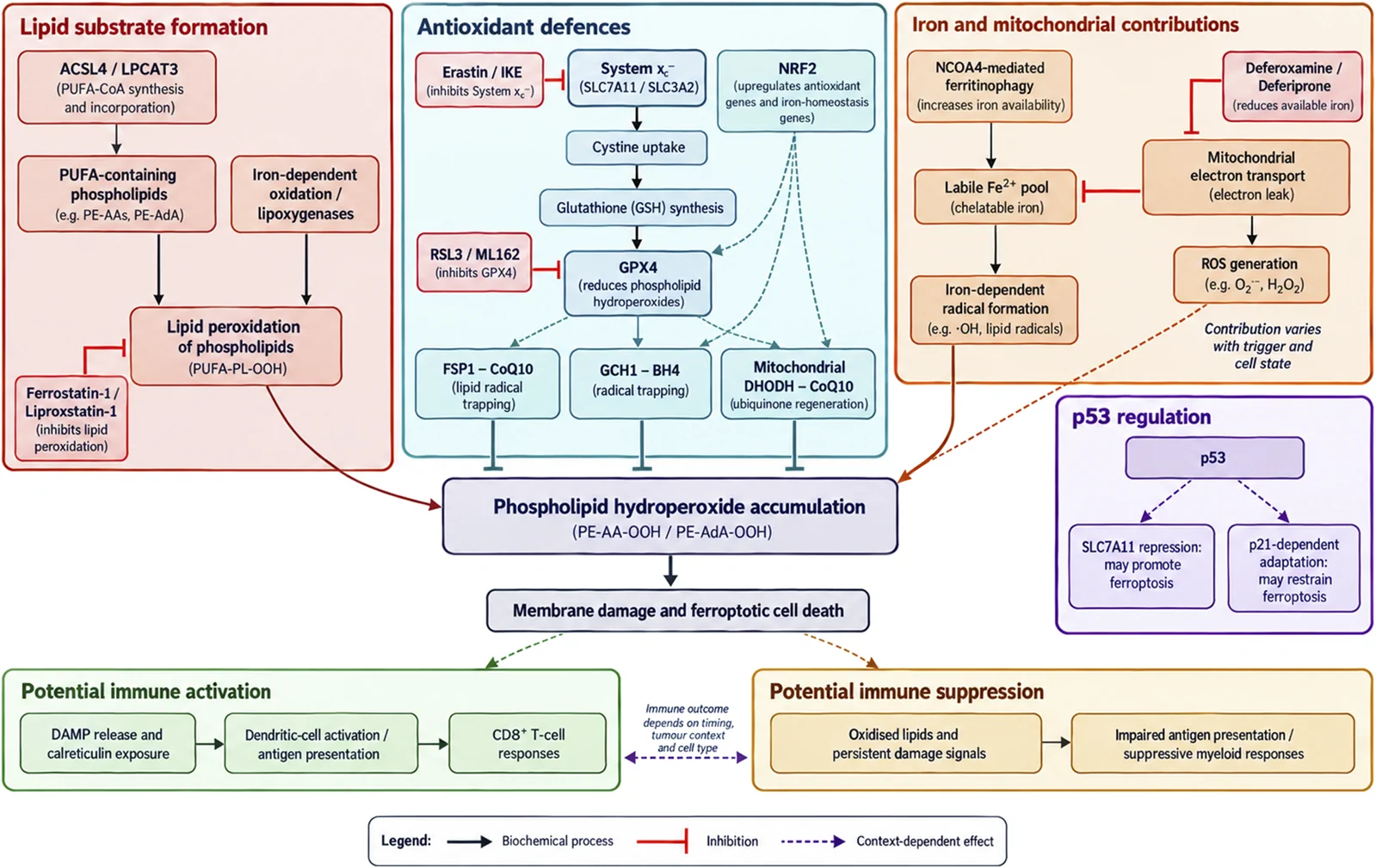
Integrated molecular pathway map of ferroptosis, mitochondrial stress and redox imbalance in precision drug therapeutics.

## Ferroptosis as a drug-responsive biochemical programme

Ferroptosis is driven by the accumulation of phospholipid hydroperoxides within cellular membranes. GPX4 remains a central ferroptosis suppressor because it detoxifies lipid hydroperoxides and preserves membrane integrity (Zhang et al., 2024). When GPX4 activity is impaired, or when glutathione supply falls below the level needed for repair, lipid peroxidation can exceed cellular tolerance. The result is membrane damage and regulated cell death rather than a vague increase in oxidative stress.

Pharmacologically, ferroptosis can be manipulated in two opposing directions. In oncology, ferroptosis induction may help to overcome apoptosis resistance, exploit altered iron metabolism and sensitise malignant cells to chemotherapy, radiotherapy, targeted therapy or immunotherapy (Diao et al., 2024; Yapici et al., 2024; Zhou et al., 2024). By contrast, ferroptosis inhibition may be protective in neurodegeneration, cardiovascular injury, renal injury, hepatic injury and toxicant-induced tissue damage (Li et al., 2024; Sinha, 2025).

This duality is the central translational challenge. A ferroptosis inducer may be desirable in tumour tissue but harmful in the kidney, liver, myocardium or nervous system. Conversely, a ferroptosis inhibitor may protect normal tissue but could weaken anticancer efficacy if used without a disease-specific rationale. A useful therapeutic strategy therefore has to ask a simple but demanding question: where, when and how strongly should ferroptosis be pushed? Framing the problem this way, rather than asking whether a compound is a generic inducer or inhibitor, is what distinguishes a precision pharmacological-toxicological approach from a purely mechanistic one. Figure 1 summarises this bidirectionality schematically, showing both the pharmacological inducers and the protective inhibitors acting at their respective molecular nodes.

### Expanding the regulatory landscape beyond GPX4, FSP1 and NRF2

Although GPX4, FSP1 and NRF2 form the most frequently discussed defence triad, a translationally credible framework must account for the wider regulatory ecosystem that determines whether a cell undergoes ferroptosis. Four emerging nodes are particularly relevant to drug discovery and safety prediction.

Dihydroorotate dehydrogenase (DHODH). DHODH, located at the outer surface of the inner mitochondrial membrane, reduces ubiquinone to ubiquinol and thereby provides a GPX4-independent, mitochondrially localised defence against lipid peroxidation. Mao et al. (2021) showed that DHODH cooperates with mitochondrial GPX4 to suppress ferroptosis, and that its inhibition (for example, by brequinar) sensitises low-GPX4 tumours to ferroptotic death. Because DHODH also sits on the pyrimidine-synthesis pathway already targeted clinically (e.g., teriflunomide, leflunomide), it offers a near-term repurposing opportunity, but its mitochondrial location also raises a clear toxicological flag for mitochondria-rich organs, a point developed mechanistically below at the level of individual electron transport chain complexes and depicted directly in Figure 1.

GCH1-BH4 axis. GTP cyclohydrolase 1 (GCH1) generates tetrahydrobiopterin (BH4), a radical-trapping antioxidant that protects phospholipids with two polyunsaturated fatty-acyl tails and helps regenerate reduced CoQ10. Soula et al. (2020) and Kraft et al. (2020) identified the GCH1–BH4 axis as a GPX4-and FSP1-independent resistance pathway. Its activity may explain why some tumours resist GPX4 inhibition, and its modulation provides an additional lever for both sensitisation and protection.

Ferritinophagy and NCOA4. The labile iron pool that fuels lipid peroxidation is replenished in part by ferritinophagy, the selective autophagic degradation of ferritin mediated by the cargo receptor NCOA4. By controlling iron release, NCOA4 sets the ceiling on Fenton-type chemistry and lipid-radical propagation (Kang et al., 2024). Pharmacologically, promoting ferritinophagy may enhance ferroptosis in iron-avid tumours, whereas limiting it may protect tissues exposed to iron-overload or ischaemia–reperfusion stress.

p53 and transcriptional control. p53 exerts context-dependent control over ferroptosis. It can promote ferroptosis by repressing SLC7A11 (the catalytic subunit of system Xc–) and can also restrain it through p21-dependent metabolic adaptation (Liu and Gu, 2022). This dual behaviour is therapeutically important because p53 status, which varies widely across tumours, may predict whether a given malignancy is sensitised or protected by a ferroptosis-directed agent. A general statement that p53 behaves in a context-dependent manner, however, gives a clinician or drug developer no operational rule to apply at the point of patient selection. Table 3 therefore converts this observation into an explicit, provisional stratification framework linking p53 status and the predictive biomarker profile in Table 2 to a recommended therapeutic action, so that context-dependence is expressed as a decision rule rather than left as an unresolved caveat.

Taken together, these regulators show that ferroptosis sensitivity is set by a distributed network rather than by GPX4 alone. A drug that engages only one node may be bypassed by another, which has direct consequences for both resistance (in oncology) and unexpected organ toxicity (in normal tissue). Table 1 maps each node onto a representative agent, its chemical and pharmacokinetic class, a therapeutic opportunity and a toxicological concern, and Figure 1 places every one of these nodes within its subcellular compartment so that the crosstalk between them—rather than each node in isolation—is visible at a glance.

### Mitochondrial biochemistry of ferroptosis: electron transport chain crosstalk and compartmentalised lipid pools

Mitochondria regulate ATP generation, reactive oxygen species production, iron metabolism, lipid oxidation and cell-death signalling, and a translationally credible account of mitochondrial stress in ferroptosis must move beyond this general statement to the level of individual electron transport chain (ETC) complexes and their distinct lipid-peroxidising consequences. Figure 1 depicts this compartment explicitly as a bounded mitochondrial region, distinguishing inner-membrane chemistry (Complex I, Complex III, DHODH, Fenton chemistry) from the outer-membrane VDAC1 node, so that the reader can trace each species-generating step without inferring it from prose alone. The mitochondrion is the principal cellular source of reactive oxygen species because electrons leaking from complex I and complex III reduce oxygen to superoxide, which superoxide dismutase converts to hydrogen peroxide (Gan, 2021; Murphy, 2009). Hydrogen peroxide reacts with ferrous iron through Fenton chemistry to generate hydroxyl radicals capable of abstracting the bis-allylic hydrogen from polyunsaturated fatty acid chains, initiating the carbon-centred radical cascade that culminates in lipid hydroperoxide formation (Gan, 2021; Murphy, 2009; Conrad and Pratt, 2019; Feng et al., 2024).

Complex I and complex III are not interchangeable in this respect. Pharmacological blockade of complex I promotes lipid peroxidation and potentiates ferroptosis in several cell systems, an effect attributed to mitophagy-dependent reactive oxygen species increases (Basit et al., 2017) and confirmed using mitochondria-targeted lipid-peroxidation probes showing that complex I-derived, but not complex III-derived, reactive oxygen species drive mitochondrial lipid peroxidation under cysteine or glutathione depletion (Lyamzaev et al., 2023). By contrast, complex III dysfunction has been separately implicated as a mitochondrial reactive oxygen species source that drives ferroptosis under cysteine deprivation or direct GPX4 inhibition, acting in part through voltage-dependent anion channel one oligomerisation at the outer mitochondrial membrane (Jang et al., 2024). Because the two complexes occupy different positions in the chain, generate superoxide on different faces of the inner membrane, and appear to dominate under different upstream triggers, an inhibitor acting on one complex cannot be assumed to reproduce the ferroptotic consequences of inhibiting the other, and mechanistic claims about mitochondrial toxicity should specify which complex, and which membrane face, is implicated.

This complex-specific behaviour is compounded by the fact that ferroptosis inducers fall into mechanistic classes that differ in whether they provoke mitochondrial, as opposed to non-mitochondrial, lipid peroxidation. System Xc–blockers and CoQ-depleting agents can induce substantial mitochondrial lipid peroxidation, whereas direct GPX4 inhibitors such as RSL3 predominantly drive non-mitochondrial peroxidation because DHODH continues to defend the mitochondrial inner membrane even when cytosolic and mitochondrial GPX4 are inactivated (Gan, 2021; Mao et al., 2021). This is the mechanistic reason why DHODH inhibition carries a disproportionate mitochondrial toxicity signal in Table 1: DHODH sits at the outer face of the inner mitochondrial membrane precisely where complex I- and complex III-derived superoxide is generated, and its inhibition removes the one defence system with no cytosolic equivalent, because the double membrane of the mitochondrion physically excludes cytosolic GPX4 and FSP1 from mitochondrial inner-membrane lipid pools (Gan, 2021). Tissues with high mitochondrial density and high oxidative-phosphorylation flux, including cardiomyocytes, hepatocytes and proximal tubular epithelium, are for this reason mechanistically distinct from tissues that rely predominantly on non-mitochondrial (plasma membrane, endoplasmic reticulum) lipid pools for their principal antioxidant defence, and this distinction should directly inform which organ-toxicity assays are prioritised for a given class of ferroptosis modulator.

The practical implication is that a mechanistic mitochondrial safety programme for ferroptosis-directed drugs cannot rely on a single generic mitochondrial toxicity assay. It requires, at minimum, complex-resolved respirometry using complex-specific substrates and inhibitors to distinguish complex I-, complex III- and DHODH-mediated liabilities; mitochondria-targeted lipid-peroxidation probes capable of distinguishing inner-membrane from cytosolic oxidation; and an explicit determination of whether a candidate compound behaves mechanistically as a complex I, complex III or DHODH-directed liability, since each predicts a different organ-toxicity signature and a different rescue strategy, for example, mitochondria-targeted antioxidants for complex I- or complex III-driven injury as opposed to coenzyme Q or ubiquinol supplementation for DHODH-driven injury.

### Lipid peroxidation is not a uniform event: substrate specificity and tissue-selective membrane vulnerability

Treating lipid peroxidation as a single, monolithic accumulation of oxidised polyunsaturated fatty acids obscures the substrate specificity that determines which tissues are vulnerable and which biomarkers are informative. ACSL4 activates arachidonic acid and adrenic acid to their coenzyme A thioesters, and LPCAT3 re-esterifies these activated fatty acids into phosphatidylethanolamine, generating the arachidonoyl- and adrenoyl-phosphatidylethanolamine species (PE-AA and PE-AdA) that are the preferred substrates for ferroptotic lipid peroxidation (Doll et al., 2017; Kagan et al., 2017). Cells and tissues with high ACSL4 and LPCAT3 expression are enriched for these PE species and are correspondingly more vulnerable to ferroptosis, while tissues with a membrane phospholipid profile weighted towards monounsaturated or saturated acyl chains are comparatively protected regardless of GPX4 activity (Doll et al., 2017; Kagan et al., 2017).

Enzymatic oxidation of PE-AA and PE-AdA is itself spatially organised: the scaffold protein PEBP1 redirects 15-lipoxygenase to generate PE hydroperoxides specifically at these di-unsaturated species, converting what would otherwise be comparatively slow non-enzymatic autoxidation into a rapid, targeted lipid-peroxidation programme (Wenzel et al., 2017). This substrate specificity has two direct translational consequences that a generic account of lipid peroxidation does not capture. First, generic readouts such as malondialdehyde and 4-hydroxynonenal, while convenient, do not distinguish ferroptosis-relevant PE-AA/PE-AdA oxidation from unrelated lipid oxidation and are best regarded as screening rather than confirmatory pharmacodynamic markers; targeted lipidomic quantification of oxidised PE-AA and PE-AdA species is the more specific tier one pharmacodynamic readout, and Table 2 has been revised accordingly. Second, tissue-level differences in ACSL4/LPCAT3 expression and membrane PUFA composition, rather than GPX4 expression alone, help to explain why organs with comparable mitochondrial density can nonetheless show divergent ferroptotic vulnerability, and baseline lipidomic phenotyping of the relevant normal tissue, alongside the tumour, should be regarded as part of the safety-stratification workup rather than an optional refinement. The ACSL4/LPCAT3-PEBP1-15-LOX axis is shown explicitly in Figure 1 as the substrate-generating arm of the cytosolic/ER compartment, feeding directly into the convergent lipid-peroxidation node alongside the mitochondrial contribution.

### Redox imbalance and antioxidant defence failure

Redox imbalance links ferroptosis to both therapeutic efficacy and toxicological injury. Under physiological conditions, glutathione, GPX4, thioredoxin, NADPH, BH4, NRF2-regulated enzymes and lipid-soluble antioxidants maintain redox homeostasis. Ferroptosis becomes more likely when this defence network is overwhelmed, genetically weakened or pharmacologically suppressed.

NRF2 is a major regulator of antioxidant defence, iron metabolism, glutathione synthesis and detoxification pathways. Yan et al. (2023) describe NRF2 as a key ferroptosis regulator because it influences iron, lipid and amino acid metabolism. The context matters. In cancer, persistent NRF2 activation can support ferroptosis resistance and drug tolerance; in normal tissues, NRF2 activation may reduce oxidative damage and preserve cellular viability.

FSP1 provides an important GPX4-independent ferroptosis defence pathway. Bersuker et al. (2019) and Doll et al. (2019) independently showed that FSP1 suppresses ferroptosis through CoQ10-dependent lipid radical trapping. This shifted ferroptosis biology beyond the canonical glutathione-GPX4 pathway and opened a more realistic view of resistance, in which cells may survive by rerouting antioxidant protection rather than simply restoring GPX4 function. The DHODH and GCH1–BH4 pathways described above extend this redundancy further, implying that durable ferroptosis induction may require combined or sequential targeting rather than single-node inhibition, a point taken up quantitatively below.

### Immunomodulatory consequences of ferroptosis in the tumour microenvironment

Ferroptotic cell death does not interact with the immune system as a generic toxicological liability; it has a distinct, kinetics-dependent immunological signature that can favour or oppose the same therapeutic goal depending on when, and how completely, it is measured. Early ferroptotic cancer cells expose calreticulin and release adenosine triphosphate and high-mobility group box one protein (HMGB1), a combination of damage-associated molecular patterns (DAMPs) that can promote dendritic cell maturation, antigen cross-presentation and CD8^+^ T-cell-mediated antitumour immunity, so that ferroptosis induction can in principle act as a form of immunogenic cell death and synergise with immune checkpoint blockade (Demuynck et al., 2021; Wang et al., 2019).

As ferroptosis progresses towards late-stage membrane rupture, however, the same cells become a source of oxidised phospholipids that impair dendritic cell phagocytosis and antigen cross-presentation, while HMGB1 and other DAMPs released in this later, more necrotic-like phase can instead recruit myeloid-derived suppressor cells and tumour-associated macrophages, promote an immunosuppressive microenvironment, and in some tumour contexts increase programmed death-ligand one expression (Catanzaro et al., 2024; Demuynck et al., 2021). The same molecular output, HMGB1 release, can therefore mark either an immunostimulatory or an immunosuppressive event depending on the stage of ferroptosis, the tumour type, and the surrounding cytokine and hypoxic milieu. Framing this immunological duality purely as a toxicological concern, as a generic mechanistic account tends to do, understates its therapeutic relevance. Figure 1 renders this bifurcation explicitly as two parallel, time-ordered branches beneath the convergent cell-death node, so that the early immunogenic and late tolerogenic outputs of the same HMGB1 signal can be compared directly.

From a translational standpoint, this argues for adding an explicit immunological dimension to the biomarker and dosing strategy set out elsewhere in this Perspective: monitoring circulating HMGB1, calreticulin exposure and tumour-infiltrating myeloid and CD8^+^ T-cell populations as pharmacodynamic-immunological co-markers alongside the lipid-peroxidation and organ-injury markers in Table 2, and favouring dosing regimens and combination timing that capture the early, immunogenic window of ferroptosis rather than driving tumours into a late-stage, tolerogenic phenotype. This immunomodulatory dimension has been added explicitly to the toxicological-concern column of Table 1 for GPX4-and system Xc-directed agents.

### Mitochondrial stress as a therapeutic and toxicological switch

In cancer, the electron transport chain crosstalk and compartmentalised lipid pools described above mean that mitochondrial stress may expose malignant cells to ferroptosis when they carry high metabolic demand, altered iron handling or reduced antioxidant reserve (Yapici et al., 2024). The same logic, however, creates risk in mitochondria-rich organs. The heart, liver, kidney and brain may be especially vulnerable when drugs disturb redox balance or lipid-repair capacity (Diao et al., 2024; Sinha, 2025). For that reason, ferroptosis-focused drug development should include the complex-resolved mitochondrial respiration assays, membrane-potential analysis and compartment-specific lipid-peroxidation profiling described above, together with organ-specific toxicity models, rather than relying only on conventional cytotoxicity readouts.

### Pharmacological opportunities across the ferroptosis-mitochondria-redox axis

The ferroptosis-mitochondrial stress-redox axis creates several pharmacological opportunities. Ferroptosis inducers may be useful in malignancies with high iron load, lipid metabolic reprogramming, therapy resistance or strong antioxidant dependency. Plausible strategies include GPX4 inhibition (e.g., RSL3, ML162), system Xc-blockade (e.g., erastin, sulfasalazine, imidazole ketone erastin), FSP1 inhibition (e.g., iFSP1), DHODH inhibition (e.g., brequinar), iron modulation, amplification of lipid peroxidation and combination treatment with chemotherapy, radiotherapy or immunotherapy (Diao et al., 2024; Mao et al., 2021; Yapici et al., 2024; Zhou et al., 2024).

Ferroptosis inhibitors, on the other hand, may have value in drug-induced liver injury, nephrotoxicity, cardiotoxicity, neurodegeneration and ischaemia–reperfusion injury, as summarised in Table 1, although, as set out below, these disease contexts differ sharply in their pharmacokinetic and scheduling requirements and should not be treated as pharmacologically interchangeable. Protective strategies include iron chelation (e.g., deferoxamine), radical-trapping antioxidants (e.g., ferrostatin-1, liproxstatin-1), NRF2 activation, mitochondrial stabilisation, glutathione restoration and lipid-peroxide detoxification (Li et al., 2024; Sinha, 2025; Zhang et al., 2024). The therapeutic decision is therefore not whether ferroptosis is good or bad, but whether its direction, intensity and delivery match the biological and pharmacokinetic setting.

### Toxicological risks and safety translation

The main toxicological risk in ferroptosis-based therapeutics is loss of selectivity. Classical ferroptosis activators may damage immune cells, haematopoietic stem cells, liver and kidney tissue when they are insufficiently selective (Diao et al., 2024; Liu et al., 2024). This concern is most acute in tissues with high mitochondrial activity, strong lipid turnover or limited antioxidant reserve, and it is amplified for mitochondrially targeted strategies such as DHODH inhibition, for the electron transport chain reasons set out above (Table 1).

Safety translation should therefore move beyond general cytotoxicity assays. Drug-development programmes should measure ferroptosis-relevant endpoints, including malondialdehyde, 4-hydroxynonenal, targeted lipidomic quantification of oxidised PE-AA/PE-AdA species, glutathione depletion, GPX4 activity, complex-resolved mitochondrial respiration, labile iron pool, lipid ROS, circulating DAMPs (HMGB1, calreticulin exposure) and rescue responses after iron chelation or lipophilic antioxidant treatment (Sinha, 2025; Zhang et al., 2024). These readouts would give developers more than a binary viability result; they would show whether a compound is killing cells through the intended biochemical route, in the intended subcellular compartment, and with the intended immunological consequence.

### Physiological and formulation barriers to ferroptosis-modulating agents

Proposing tumour-selective nanocarriers and microenvironment-activated prodrugs as a general solution to the selectivity problem understates the specific physicochemical and physiological barriers that these formulation strategies must overcome, and these barriers differ by chemical class rather than being generic. Most tool-compound ferroptosis inducers and inhibitors, including RSL3, ML162, erastin, imidazole ketone erastin and the radical-trapping antioxidants ferrostatin-1 and liproxstatin-1, are highly lipophilic small molecules whose poor aqueous solubility, rather than a simple lack of tumour targeting, is the primary reason for their limited pharmacokinetic and clinical tractability (Zaffaroni and Beretta, 2021).

Encapsulation in liposomes, polymeric nanoparticles or lipid nanoparticles can improve solubility and circulation time, but each strategy introduces its own liability. Nanoparticle constructs are typically restricted to the tumour vasculature and interstitium by enhanced permeability and retention, an effect that is heterogeneous across tumour types and frequently reduced, rather than enhanced, in poorly vascularised or fibrotic tumours. Iron-oxide-based nanocarriers, attractive for magnetically targeted ferroptosis induction, introduce an exogenous iron load that can itself perturb the labile iron pool and confound interpretation of whether ferroptosis is being driven by the payload or by the carrier. Antibody-drug conjugate and prodrug strategies that rely on activation by low pH or high glutathione turnover in the tumour microenvironment depend on a degree of microenvironmental heterogeneity that is itself variable between, and within, tumours, so activation cannot be assumed to be uniform across a lesion. For agents intended to act in the central nervous system, such as ferroptosis inhibitors proposed for neurodegeneration, the additional requirement of blood-brain-barrier permeability substantially narrows the field of deliverable chemistries and is rarely satisfied by the nanocarrier platforms developed for peripheral tumour delivery.

Delivery strategy should therefore be selected to match the specific physicochemical liability of the chemical class in question, as set out in the pharmacokinetic classification added to Table 1, rather than treated as a single generic engineering solution applicable across all ferroptosis-modulating chemotypes.

### Precision therapeutics and biomarker-guided stratification

A major limitation in ferroptosis translation is the lack of validated clinical biomarker panels. Ferroptosis is often inferred from lipid peroxidation, iron accumulation or antioxidant depletion, yet no single marker is sufficient and most candidates remain at the preclinical or exploratory stage. A precision framework should therefore combine biochemical, molecular and functional indicators across three tiers: tissue-based predictive markers that estimate intrinsic ferroptosis sensitivity, dynamic pharmacodynamic markers that confirm on-target engagement, and circulating safety markers that flag off-target organ injury early. We add to this an explicit, provisional set of benchmark values in Table 2, offered as a hypothesis-generating starting point for prospective validation rather than as established clinical thresholds, in direct response to the observation that a panel is of limited practical use if it specifies which markers to measure without indicating what an informative result would look like.

Relevant biomarker domains include GPX4 expression, ACSL4 expression, SLC7A11/system Xc–activity, glutathione status, NRF2 activation, FSP1 expression, DHODH and GCH1-BH4 activity, CoQ10 metabolism, oxidised PE-AA/PE-AdA burden, mitochondrial respiratory reserve, iron-handling proteins and circulating DAMPs (Dai et al., 2024; Zhou et al., 2024). In oncology, these markers may help to identify ferroptosis-sensitive tumours; ACSL4, which incorporates polyunsaturated fatty acids into membrane phospholipids, is among the most promising predictive candidates because high ACSL4 expression repeatedly tracks with ferroptosis sensitivity (Doll et al., 2017). In toxicology, the same panel may help to identify patients at higher risk of drug-induced oxidative organ injury. Patient stratification would then proceed by combining a predictive tissue signature (e.g., high ACSL4 with low GPX4/SLC7A11) with an assessment of organ-specific reserve (baseline glutathione, iron status, and mitochondrial, renal and hepatic function), so that inducers are directed towards sensitive tumours in patients with adequate normal-tissue protection. We summarise a proposed, tiered candidate panel, its current validation status and provisional benchmarks in Table 2, and we convert the p53 and predictive-tier findings into an explicit stratification rule in Table 3.

### A conceptual pharmacokinetic-pharmacodynamic framework for overlapping therapeutic and toxic exposure windows

The central pharmacological problem identified throughout this Perspective, namely, that the same biochemical pathway can be therapeutic in tumour tissue and toxic in normal tissue, can be given an explicit, if necessarily provisional, quantitative structure. If tumour ferroptotic efficacy and normal-tissue ferroptotic toxicity are each modelled as sigmoidal exposure-response relationships of the classical form Effect = Emax × C^n^/(EC50^n^ + C^n^), where C is the local tissue exposure, approximated by the area under the concentration-time curve (AUC) in that tissue, and EC50 is the exposure producing half-maximal effect, then a provisional therapeutic index for a ferroptosis-directed agent can be defined as the ratio of the organ-toxicity EC50 (or a conservative threshold such as the exposure producing ten per cent of maximal toxic effect) to the tumour-efficacy EC50 in the same patient or model.

A therapeutic index appreciably greater than one indicates an exploitable window in which tumour engagement can be achieved before normal-tissue toxicity thresholds are reached. A therapeutic index close to or below one indicates that efficacy and toxicity are essentially co-incident and that dose escalation alone cannot separate them, in which case tumour-selective delivery, biomarker-guided patient selection, or a shift to a different node in the network, for example, combining a sub-toxic dose of a DHODH inhibitor with a tumour-restricted GPX4 inhibitor rather than using either agent alone at a fully effective dose, becomes the principal route to a usable window.

Because mitochondrial density, DHODH expression and baseline glutathione reserve differ systematically between organs, the organ-specific EC50 for toxicity is expected to vary by tissue for the same systemic exposure, and a population pharmacokinetic model for this drug class should therefore include organ-specific distribution or sensitivity terms rather than a single systemic exposure-toxicity relationship. We propose this Emax-based framework as a conceptual scaffold for prospective preclinical work, in which paired tumour-organoid or patient-derived xenograft efficacy curves and isolated-organ or isolated-mitochondria toxicity curves are generated across a shared concentration range for each candidate agent, allowing the therapeutic index to be estimated before first-in-human dosing rather than inferred retrospectively from adverse events. This framework does not replace, and is not itself, quantitative pharmacokinetic data; rather, it specifies the minimum paired-curve experimental design that would allow such data to be generated and interpreted consistently across the agent classes in Table 1.

### Acute versus chronic disease contexts: divergent scheduling and delivery requirements

Grouping ischaemia-reperfusion injury, acute kidney injury and drug-induced cardiotoxicity together with neurodegenerative disease under a single ferroptosis-inhibition umbrella, as is common in the literature, obscures pharmacokinetic and scheduling requirements that differ as much between these conditions as ferroptosis induction differs from ferroptosis inhibition. Ischaemia-reperfusion injury, acute kidney injury and acute drug-induced cardiotoxicity are time-limited insults with a defined peri-injury window, typically hours, during which a ferroptosis inhibitor needs to achieve adequate peripheral organ exposure for a short, defined period. Systemic, non-blood-brain-barrier-penetrant agents such as deferoxamine or ferrostatin-class radical-trapping antioxidants are pharmacologically well matched to this context, provided they can be delivered rapidly enough, for example, peri-procedurally, before reperfusion, or before or during nephrotoxic or cardiotoxic drug administration, and provided iron chelation is not sustained long enough to produce systemic iron deficiency.

Neurodegenerative disease, in contrast, requires chronic, often lifelong, ferroptosis suppression in a compartment protected by the blood-brain barrier, which excludes most peripherally effective radical-trapping antioxidants and iron chelators at tolerable systemic doses and instead requires chemistries specifically selected or engineered for central nervous system penetrance, together with long-term monitoring for cumulative toxicity, including chronic iron-chelation effects and off-target central nervous system exposure, that is simply not relevant to a single peri-procedural dose.

Treating these as a single therapeutic category risks two distinct errors: applying acute, short-exposure dosing logic to a chronic neurodegenerative context, where it will be inadequate, or applying chronic dosing and blood-brain-barrier-penetrant chemistry requirements to an acute peripheral injury, where they are unnecessary and would needlessly restrict the usable chemical space. Protocol and formulation decisions for ferroptosis-inhibiting agents should therefore be organised primarily around this acute-versus-chronic and peripheral-versus-central axis, with the specific organ (kidney, heart, liver, brain) treated as a secondary, rather than primary, axis of classification.

## Research priorities

Future research should first standardise and validate ferroptosis biomarkers across disease contexts, because current studies often use overlapping but non-equivalent markers and inconsistent thresholds. Prospective qualification of the tiered panel and provisional benchmarks proposed in Table 2, including defined cut-offs and assay harmonisation, is a priority, as is prospective testing of the p53-and biomarker-guided decision rules set out in Table 3. Complex-resolved mitochondrial assays, capable of distinguishing complex I-, complex III- and DHODH-mediated liabilities, should be integrated into ferroptosis drug screening, particularly for compounds intended for chronic use, for mitochondrially targeted strategies such as DHODH inhibition, or for patients with pre-existing metabolic vulnerability. Lipidomic technologies should be used to quantify disease-specific oxidised PE-AA/PE-AdA signatures rather than treating lipid peroxidation as a single generic endpoint, and immunological pharmacodynamic monitoring (circulating HMGB1, calreticulin exposure, tumour-infiltrating myeloid and CD8^+^ T-cell populations) should be incorporated into early-phase trials to distinguish immunogenic from tolerogenic ferroptotic responses.

A further priority is pharmacokinetic-pharmacodynamic modelling of the kind outlined above: paired efficacy and toxicity exposure-response curves should be generated for each candidate agent to estimate a provisional therapeutic index before first-in-human dosing, with organ-specific distribution or sensitivity terms built into population pharmacokinetic models. Drug-delivery research should advance tumour-selective nanocarriers and microenvironment-activated prodrugs matched to the specific physicochemical liability of each chemical class, and should develop blood-brain-barrier-penetrant chemistries separately from peripheral formulations rather than assuming interchangeability. Clinical trials should stratify patients by the p53-and biomarker-guided framework in Table 3, by mitochondrial vulnerability, and by organ-specific risk, and should explicitly separate acute, short-exposure trial arms (ischaemia-reperfusion injury, acute kidney injury, acute cardiotoxicity) from chronic, central-nervous-system-directed arms (neurodegeneration), each with its own delivery chemistry and monitoring schedule. Regulatory toxicology should consider ferroptosis-specific endpoints, including compartment-resolved mitochondrial and lipidomic markers, for drugs that alter redox, iron or mitochondrial pathways (Dai et al., 2024; Sinha, 2025).

## Discussion

Ferroptosis has moved from a mechanistic cell-death concept to a major translational theme in pharmacology and toxicology. Its value lies in its ability to connect biochemical processes that are central to drug action: iron metabolism, lipid peroxidation, mitochondrial stress, glutathione depletion, antioxidant defence and membrane injury. The contribution of the present framework is to insist that these processes be read in two directions at once, as sources of both efficacy and toxicity, and to resolve each process to the level of mechanistic and quantitative detail needed for translation: individual electron transport chain complexes rather than mitochondrial stress in general, specific oxidised phospholipid species rather than lipid peroxidation in general, a kinetics-dependent immunological output rather than a single toxicological label, and an explicit pharmacokinetic-pharmacodynamic structure rather than a qualitative efficacy-toxicity trade-off. Concrete tools are provided for doing so (Tables 1-3; Figure 1), and Figure 1 in particular is intended to let a reader trace, in a single view, how a perturbation entering at any one node a drug, a genetic lesion, or an environmental insult propagates through the oxidative-stress, mitochondrial, lipid-substrate and immunological layers described in the text.

The expanded mechanistic landscape reinforces this view. Because DHODH, GCH1–BH4, NCOA4-ferritinophagy and p53 each provide independent control over ferroptosis sensitivity, and because complex I and complex III contribute to mitochondrial lipid peroxidation through distinct mechanisms, single-node strategies are vulnerable to compensatory resistance in tumours and to unpredictable injury in normal tissue. This redundancy is precisely why biomarker-guided stratification and combination logic, rather than blanket induction or inhibition, are likely to define successful translation, and why the p53-guided decision framework in Table 3 is offered as an explicit, testable rule rather than a restatement of context-dependence.

Even so, enthusiasm needs to be tempered by caution. Ferroptosis is not inherently beneficial or harmful. Its value depends on whether it occurs in the intended tissue, at the intended intensity, in the intended subcellular compartment, with the intended immunological consequence, and within a controllable therapeutic window as defined by the exposure-response relationships outlined above. In cancer, ferroptosis induction may be desirable. In ischaemia-reperfusion injury and acute drug-induced organ toxicity, ferroptosis suppression delivered within a short peri-injury window may be protective; in neurodegeneration, the same protective goal requires a chronically administered, blood-brain-barrier-penetrant chemistry that is pharmacologically distinct from the acute case (Chen et al., 2024; Diao et al., 2024; Sinha, 2025). This is the central argument of the framework: therapeutic precision depends on biochemical, compartmental, immunological and pharmacokinetic context, not on treating ferroptosis as a universally favourable or unfavourable pathway, nor on treating any single disease category as pharmacologically uniform.

## Conclusion

Ferroptosis, mitochondrial stress and redox imbalance represent a powerful but complex axis in modern drug therapeutics. Their integration offers new opportunities for treating resistant disease, predicting organ toxicity and designing precision interventions. The future of ferroptosis-based therapeutics will depend on selective targeting informed by the full regulatory network, including DHODH, GCH1–BH4, NCOA4 and p53, resolved at the level of individual electron transport chain complexes and specific oxidised phospholipid species and consolidated in a single, comprehensive pathway diagram (Figure 1); validated and tiered biomarkers with explicit provisional benchmarks; a p53-and biomarker-guided stratification rule; mitochondrial safety assessment; delivery platforms matched to the physicochemical liability of each chemical class; explicit pharmacokinetic-pharmacodynamic modelling of overlapping therapeutic and toxic exposure windows; a clear separation of acute from chronic disease scheduling requirements; and a clear distinction between therapeutic cell death and toxicological injury, including its immunological dimension. A pharmacological-toxicological biochemistry framework of this resolution provides a rational foundation for translating ferroptosis biology into safer, more effective and more precise drug therapeutics.

Drug exposure may influence mitochondrial respiration, resolved here at the level of complex I- and complex III-derived superoxide and DHODH-dependent defence, iron metabolism and NCOA4-mediated ferritinophagy, glutathione availability, GPX4 activity, FSP1-CoQ10 and GCH1–BH4 signalling, NRF2-regulated antioxidant defence, p53-dependent control of SLC7A11, ACSL4/LPCAT3-directed generation of oxidisable PE-AA/PE-AdA species, and overall lipid-peroxidation burden. The diagram is organised into three bounded subcellular compartments (cytosol/plasma and endoplasmic-reticulum membrane; mitochondrion, with the inner-membrane and outer-membrane VDAC1 nodes distinguished; and nucleus) that converge on a single lipid-peroxidation node, so that the cross-talk between compartments requested for a comprehensive pathophysiological view is shown directly rather than left to be inferred from separate sections of text. Representative inducers (e.g., RSL3, erastin, brequinar, iFSP1) are shown as red blockade arrows terminating at their respective defence nodes, and representative protectors (e.g., ferrostatin-1, deferoxamine, bardoxolone) are shown as blue arrows converging on the same nodes from the opposite direction, so that drug responsiveness at each mechanistic step is depicted alongside the underlying biochemistry rather than described only in Table 1.

Downstream of membrane rupture, an early, immunogenic phase (calreticulin exposure, ATP and HMGB1 release, dendritic-cell maturation and CD8^+^ T-cell-mediated antitumour immunity) is shown as a distinct, time-ordered branch from a later, more tolerogenic phase (oxidised phospholipids, impaired antigen cross-presentation, myeloid-derived suppressor cell and tumour-associated macrophage recruitment, and PD-L1 upregulation), reflecting the kinetics-dependent immunological duality described in the main text. Biomarker-guided stratification using the tiered panel and provisional benchmarks (Table 2), the p53-guided decision rule (Table 3), mitochondrial-function testing, lipid-peroxide monitoring, tumour-selective delivery matched to chemical class, and organ-specific and acute-versus-chronic safety assessment remain the translational requirements for precision ferroptosis-based therapeutics; Figure 1 is intended as the single schematic reference against which each of these requirements can be located mechanistically.

## Data Availability

The original contributions presented in the study are included in the article/supplementary material, further inquiries can be directed to the corresponding author.
